# Pharmacological Effect of Quercetin in Hypertension and Its Potential Application in Pregnancy-Induced Hypertension: Review of* In Vitro*,* In Vivo*, and Clinical Studies

**DOI:** 10.1155/2018/7421489

**Published:** 2018-12-02

**Authors:** Marcin Ożarowski, Przemysław Ł. Mikołajczak, Radosław Kujawski, Karolina Wielgus, Andrzej Klejewski, Hubert Wolski, Agnieszka Seremak-Mrozikiewicz

**Affiliations:** ^1^Institute of Natural Fibres and Medicinal Plants, Wojska Polskiego 71b, 60-630 Poznań, Poland; ^2^Department of Pharmacology, Poznań University of Medical Sciences, Rokietnicka, 5a, 60-806 Poznań, Poland; ^3^Department of Nursing, Poznan University of Medical Sciences, Poznań, Poland; ^4^Department of Obstetrics and Women's Diseases, Poznan University of Medical Sciences, Poznań, Poland; ^5^Department of Perinatology and Women's Diseases, Poznan University of Medical Sciences, Polna 33, 60-535 Poznań, Poland; ^6^Division of Gynecology and Obstetrics, Podhale Multidisciplinary Hospital, Nowy Targ, Poland; ^7^Laboratory of Molecular Biology, Poznan University of Medical Sciences, Polna 33, 60-535 Poznań, Poland

## Abstract

Since improving maternal and child health is a public health priority worldwide, the main aim of treatment of hypertension in pregnant women is to prevent complications during pregnancy, labor, and postpartum. In consequence, much attention is paid to the use of antihypertensive drugs that can be used safely during pregnancy. Several side effects of methyldopa, which is currently the most commonly used antihypertensive drug in pregnant women, mean that the search for an effective and safe alternative still continues. Flavonoid compounds present in medicinal plants, vegetables, and fruits may be a promising source of new drugs. In this aspect, quercetin, a well-known flavonoid due to its antihypertensive action, may be considered a prototype for safe antihypertensive drugs. This review focuses on the selective activity of quercetin. Based on recent studies, a few problems were discussed, including (1) pathology of pregnancy-induced hypertension; (2) search for new pharmacological treatments of pregnancy-induced hypertension; (3) issues with the use of herbal extracts during pregnancy; (4) flavonoids as natural active chemical compounds; (5) quercetin: its action during pregnancy,* in vitro *and* in vivo* pharmacological activities, clinical trials, and meta-analysis; (6) quercetin intake during pregnancy; (7) other natural compounds tested during pregnancy; (8) potential problems with the use of quercetin; (9) safety profile of quercetin. Various studies have shown a beneficial effect of quercetin on vascular endothelial function and its antioxidative and anti-inflammatory activity on cellular and tissue level. It is known that in animal models quercetin affects positively the development of embryo, fetus, and placenta. Because this flavonoid did not have teratogenic and abortive effect, it is generally recognized as safe. For this reason it should be appreciated and studied in the aspect of its potential use in the prevention and treatment of pregnancy-induced hypertension among women in this risk group.

## 1. Pathology of Pregnancy-Induced Hypertension

The increasing number of patients suffering from cardiovascular diseases, including hypertension being often developed during pregnancy, indicates the need for innovative strategies for more effective prevention and treatment [1].

Hypertensive disorders during pregnancy are classified into 4 categories, as per National High Blood Pressure Education Program Working Group on High Blood Pressure in Pregnancy: (1) chronic hypertension, (2) preeclampsia-eclampsia, (3) preeclampsia superimposed on chronic hypertension, and (4) gestational hypertension (transient hypertension of pregnancy or chronic hypertension identified in the latter half of pregnancy) [1]. This terminology is more precise and thus it is preferred over the older yet more commonly used term pregnancy-induced hypertension (PIH) [2].

Preeclampsia (ICD-10 code 014) is defined as a complication of pregnancy characterized by a complex of symptoms including maternal hypertension and proteinuria with or without pathological edema. Preeclampsia is the most dangerous complication during pregnancy because of hypertrophy and premature detachment of the placenta, and intrauterine fetal death. Preeclampsia usually occurs after the 20th week of gestation, but may develop before this time in the presence of trophoblastic disease [3]. In detail, preeclampsia was defined as high blood pressure (≥140 mmHg systolic or ≥90 mmHg diastolic, or increases of 30 mmHg systolic or 15 mmHg diastolic from the baseline on at least two occasions, six or more hours apart) that develops from the 20th gestational week in a previously normotensive woman, with proteinuria (one 24-hour urine collection with a total protein excretion of at least 300 mg/l) [4, 5]. Preeclampsia is one of the major causes of maternal and fetal mortality worldwide with more than 75,000 maternal death cases per year [6–8]. Complications of severe hypertension in pregnant women may include intracerebral bleeding, cardiac problems, and placental dysfunction causing several other problems [9]. The etiology of preeclampsia remains yet not fully known. One of the hypotheses concerns placental endothelial cells dysfunction [10]. Changes in uteroplacental unit resulting in PIH may be also caused by the influence of renin-angiotensin system disturbances, activity of coagulation cascade elements, influence of oxidative stress, or improper inflammatory reactions [11]. Hence, the attention is focused on the endothelial damage of spiral arteries leading to the decreased concentration of vasodilators (prostacyclin, nitric oxide) and increased level of vasoconstrictors (thromboxane, endothelin-1), as well as on the explanation of impaired function of placental endothelial nitric oxide synthase.

Proper blood flow in the uteroplacental unit is a result of the presence of vasoactive humoral factors, such as nitric oxide, a crucial regulator of the flow in the uteroplacental unit [11, 12]. In case of PIH, a very low nitric oxide production leads to impaired vasodilatation. Thus, PIH development is probably due to imbalance between placental vasoconstrictors and vasodilators [13]. Endothelial nitric synthase expressed and localized in uteroplacental endothelium and trophoblasts is suggested to play an important role in the regulation of maternal and fetoplacental hemodynamics during pregnancy. The interaction between nitric oxide, forms of nitric oxide synthase, and endothelin-1 in fetoplacental unit is strongly associated with and regulates placental blood flow in normal pregnancies and was found to be disturbed in hypertensive women [14]. Any changes in endothelin-1 activity or quantity may influence the proper development of fetoplacental unit leading to PIH. Its pathogenesis is explained in such case by endothelium damage of spiral arteries during trophoblast invasion to the vessel wall [15, 16]. In PIH trophoblast invasion is limited only to decidual part of spiral arteries leading to placental anoxia and endothelial damage. These changes are evoked by the imbalance of vasodilators and vasoconstrictors. During such pathological process plasma levels of thromboxane A2 and endothelin-1 are observed to increase while nitric oxide levels are observed to decrease. The impaired balance between vasoactive factors, especially disturbances in endothelin-1 that is secreted early in pregnancy, intensifies during the second half of pregnancy [17–19]. Recently, multiple studies report that endothelin-1 levels are increased in PIH and that there is a positive correlation between endothelin-1 and severity of symptoms [20–22].

Pregnancy-induced hypertension is a state of extremely increased oxidative stress due to the decrease of antioxidant capacity. Oxidative stress as an imbalance between free radical generation and antioxidant defense is a recognized key factor in the pathogenesis of many diseases including obstetrical complications. One of the consequences of increased oxidative stress is impairment of placental blood flow, intrauterine hypoxia of the fetus, and disturbance in transfer of oxygen [44]. It is worth remarking that PIH is a multisystem disorder, which involves not only altered homeostasis of oxidants-antioxidants but also inflammatory process and endothelial dysfunction. Moreover, women are more likely to have increased oxidative stress during pregnancy, which may lead to adverse neonatal outcomes [45].

The etiology of PIH is believed to be associated with a systemic inflammatory response but few inflammatory markers are currently available to predict that state. It is suggested that women display an exaggerated inflammatory response in the course of pregnancy due to an unbalanced regulation of innate and adaptive immune responses. It is also thought that dysregulation of endogenous protective pathways might be associated with PIH etiology. Inflammation is an active process regulated by various mediators that control key cellular events to restore tissue homeostasis. Impaired resolution of inflammation probably plays vital role in the development of chronic inflammatory diseases and PIH is believed to be one of them [46].

In recent years, there has been progress in the knowledge of molecular mechanisms responsible for cellular and tissue-specific processes during pregnancy-induced hypertension. In addition to the aforementioned factors, some specific mediators that play a significant role have been identified; PIH is associated with impaired perfusion of placenta, which leads to altered secretion of markers of angiogenesis [8]. Change in endometrial levels of many angiogenic growth factors has been observed in PIH, including vascular endothelial growth factor A (VEGF-A), mRNAs encoding VEGF-C, placental growth factor (PlGF), the angiopoietins, angiopoietin 1(Ang1) and Ang2, and the receptors VEGFR-3 (Flt-4), Tie 1, Tie 2 [47, 48]. Finally, it was shown in experimental animal models of PIH that the placental growth factor (PlGF) is both a biomarker and a potential therapeutic option. PlGF is a member of the vascular endothelial growth factor (VEGF) family, is predominantly expressed in the placenta, and plays a key role in the promotion of development and maturation of placental vascular system. It is known that serum and urinary concentration of PlGF is decreased in women with preeclampsia [49, 50]. Hentschke et al. [50] observed a 62% reduction of PlGF levels in pregnant women with PIH. Rocha et al. [51] showed that low maternal plasma PlGF was associated with earlier delivery and adverse pregnancy outcomes while levels below < 40–50 pg/ml were associated with exceptionally high risk of intrauterine fetal death.

Other growth factors also play a role. It has been demonstrated by Zhou et al. [47] that several VEGF family members regulate cytotrophoblast survival and that expression of a subset of family members is dysregulated in severe forms of PIH. Relevant to PIH are also tumor necrosis factor alpha (TNF-alpha) stimulated by TNF-alpha receptor (TNFR1) (and by conditions of depleted oxygen) [52], epidermal growth factor (EGF), platelet-derived growth factor (PDGF), or insulin-like growth factor-1 (IGF-1) [49, 53, 54]. Previously, it was observed that elevated serum concentration of basic fibroblast growth factor (bFGF) was associated with mild hypertensive disorders in pregnancy [55]. Further studies have confirmed the participation of growth factors in the pathogenesis of PIH [8, 56, 57] and in the etiology of intrauterine growth restriction [58].

## 2. Searching for New Pharmacological Strategies for Pregnancy-Induced Hypertension

The main aim of treatment of hypertension in pregnant women is to prevent complications throughout pregnancy, during childbirth and postpartum. Much attention is paid to the use of antihypertensive drugs from across different pharmacological groups. Yet, from pharmacokinetic and pharmacodynamic point of view, we should be aware of special conditions for drugs use during pregnancy, namely, the presence of an additional compartment, i.e., the fetal-placental unit [9, 59]. Therefore, the impact of drugs on the fetus must be considered.

Drug treatment options in PIH are limited because some antihypertensive drugs, such as ACE inhibitors and angiotensin II receptor (AT1) antagonists (sartans), showed teratogenic effects. Methyldopa belongs to first-line treatment of PIH. It is taken per os and is well absorbed. It crosses the placenta reaching fetal serum concentrations similar to those in the mother. The onset of action is delayed for 1-2 hours after intravenous administration and 4–6 hours after oral administration; it is effective for 6–12 hours [9]. Methyldopa belongs to the group of medicines acting on central nervous system to evoke depression in the cardiovascular system [60]. Numerous reports have confirmed the high usefulness of this drug for reducing blood pressure in pregnant women, both with chronic hypertension and pregnancy-induced hypertension [60]. The reduction of blood pressure in pregnant women with hypertension using methyldopa can have a significant positive impact on the uteroplacental circulation, which improves the provision of oxygen and nutrients to the developing fetus [59, 61, 62].

Many other groups are used in the treatment of PIH, including beta blockers like metoprolol or labetalol, dihydropyridine derivatives like nitrendipine or nifedipine, and other vasodilators such as hydralazine and dihydralazine. Yet, methyldopa remains a mainstay of PIH treatment mostly due to reported uteroplacental perfusion stability and fetal hemodynamics [63, 64]. However, treatment of PIH remains challenging since methyldopa has some adverse side effects, such as hepatotoxicity [65], and may be hard to tolerate for evoking dizziness, depression, headache, etc. [66]. Generally, the use of methyldopa and labetalol has increased in recent years while the use of other antihypertensive drugs has declined [64]. Most importantly, angiotensin-converting enzyme (ACE) inhibitors as well as angiotensin-receptor blockers should be avoided in the second and third trimesters of pregnancy because of their adverse effects on fetal development. Pregnant women treated with ACE inhibitors often develop oligohydramnios, which may be caused by decreased fetal renal function [5, 63]. Also diuretics are inappropriate for their lack of efficacy in the prevention of PIH and for the risk of more nausea and vomiting [67]. For this reason, quercetin may be considered as a potential alternative method in prevention and treatment of pregnant-induced hypertension. More importantly, quercetin may have a potential application as an adjuvant therapy together with methyldopa to increase effectiveness of therapy. Therefore, administration of quercetin may be complementary and provide support for patients treated with methyldopa. This way doses of methyldopa, hence its adverse effects, could be diminished. By analogue, similar result could be expected from combining quercetin with other antihypertensive drugs (i.e., nitrendipine, nifedipine, labetalol, metoprolol, hydralazine and dihydralazine). However, additional studies should be carried out to provide evidence of such positive pharmacological interaction. The search for active substances derived from vegetables and medicinal plants is well-established in science since many medicinal discoveries come from natural sources. One of the sources of new drugs is medicinal plants, which are known to contain flavonoids with a proven antihypertensive effect [68, 69].

## 3. The Issue of the Use of Herbal Extracts during Pregnancy

Application of herbal products is one of the methods in complementary and alternative medicine [70]. Recently, several multinational studies have been carried out to determine prevalence of herbal medicine use in pregnancy. These studies allow us to deduce that an increasing number of women put their own and their unborn children's health at risk due to lack of knowledge about phytochemical properties or adequate use of herbal medicinal products, as well as lack of communication with healthcare provider [71–74]. There has been a continuous increase of interest in the use of herbal products but the available recommendations are very often controversial or contradictory [9]. The prevalence of herbal medicine use in pregnancy ranges from 7% to 55% across different geographical, social, and cultural settings and is different across ethnic groups [70, 75]. Herbal products are very often preferred over prescription medications due to the belief that herbs are safer for the fetus than modern medicine [71, 72]. Nonetheless, according to Bruno et al. [76], because the side effects and teratogenic potential of most herbal medicines are poorly understood, herbal dietary supplements are often mistaken as harmless unless proven otherwise. It is well known that plant extracts and other herbal products are complex mixtures, and their action is a result of summation of activities of several chemical components. It should be stressed that standardization of the extract does not guarantee safety of use during pregnancy [76]. It is due to the fact that while the main compound has no side effect on the fetus, often the other adjuncts to the herbal product mixture, although occurring in small amounts, may adversely affect the developing fetus. Moreover, it should be noted that plants interact with their environment. Thus, plants' chemical composition may be quite diverse [77]. Also, manufacturing process of herbal medicinal products is very complex because it encompasses nonstandardized processes like cultivation of plants, obtaining vegetable raw material from various parts of the world, preparing of extract, and producing a product in accordance with local guidelines of good manufacturing practice. It is known that each stage of such herbal drug development may affect the final composition of the herbal medicine, which is of paramount importance in light of safety of use in pregnant women [77]. Although there is clinical evidence of safe use of certain plant extracts during pregnancy, clinical data are often inconclusive due to small sample sizes, ethical reservations, or incomplete study designs [76]. Therefore, it can be concluded that using pure chemical compounds of natural origin (for example flavonoids) would be a far safer option for pregnancy and for the developing embryo or fetus since pure compounds do not contain additional substances and their precise dosage is easier. Furthermore, application of a single flavonoid allows straightforward studying of its mechanism of action and its influence on various targets during pathological processes in pregnancy.

Epidemiological and intervention studies strongly suggest that flavonoid intake has beneficial effects on vascular health [78]. Several flavonoids were investigated in various models of vascular disorders showing their antioxidant properties and vasodilator effect, as well as anti-inflammatory, antiatherogenic, and antithrombotic effect [79–82]. However, evaluation of the vascular function of dietary flavonoids remains difficult due to lack of information on their bioavailability, biotransformation, or bioaccumulation [78], especially during pregnancy. Moreover, biodynamics in the vascular system during pregnancy should be taken into account to estimate the antihypertensive effect of dietary flavonoids. Generally, the use of natural substances is a scientific and therapeutic challenge due to specific physiological and pathological changes occurring during pregnancy.

## 4. Flavonoids as Active Natural Chemical Compounds

Plant flavonoids and their conjugates belong to a large family of natural phenylpropanoid-derived polyphenolic compounds that are commonly found in many species of medicinal plants from different families, i.e., Asteraceae, Lamiaceae, Rutaceae, and Passifloraceae [83, 84], with an estimated 10,000 species [85]. Structurally, they consist of two main groups: the 2-phenylchromans (flavonoids, including flavanones, flavones, flavonols, flavan-3-ols, and anthocyanidins) and the 3-phenylchromans (isoflavonoids, including isoflavones, isoflavans, and pterocarpans) [85]. Flavonoids have been extremely important for humanity. They have been strongly linked to health benefits of beverages like tea and wine, foods like fruits and vegetables, and extracts from medicinal plants [86].

The biological activity of flavonoids has a broad spectrum and thus they have been used extensively for their anti-inflammatory, antilipidemic, antihyperglycemic, antiviral, hepatoprotective, gastric antiulcer, cardioprotective, neuroprotective, antioxidant, and anticancer properties [77]. Many studies have demonstrated a vast array of biochemical actions of flavonoids but the best-described property is their ability to act as antioxidants. It is well known that the antioxidant activity of flavonoids depends on the arrangement of functional groups with respect to the nuclear structure [84]. The protective effects of flavonoids are connected not only with their antioxidant but also vasodilatory actions [84]. Vascular endothelial function is considered as a marker in many cardiovascular problems, including hypertension [87, 88]. Endothelium is known to regulate vascular tone by balancing vasoconstriction with vasodilation to provide adequate perfusion pressure to target organs. Moreover, endothelium interacts with leukocytes, thrombocytes, and vessel wall. It also synthesizes growth factors and thromboregulatory molecules and responds to physical and chemical signals [89]. Also, endothelium influences hemostatic balance between thrombosis and anticoagulation. Other functions include regulation of angiogenesis, wound healing, smooth muscle cell proliferation, fibrosis, and inflammation [88]. Therefore, endothelial dysfunction seems to have a complex relationship with hypertension preceding the development of pathological disorders in the cardiovascular system [87] while improvements in endothelial function could contribute to lower blood pressure. Not only numerous pharmacological studies but also clinical and epidemiological investigations have shown an inverse correlation between dietary flavonoid consumption and chronic degenerative diseases, such as cardiovascular diseases (CVD) [70, 90, 91]. Results of systematic reviews and meta-analysis of prospective cohort studies have shown that a high flavonoid intake is associated with lower mortality from CVD. These results are consistent with current recommendations for a healthy diet to increase consumption of fruits and vegetables, which contain meaningful level of flavonoids [91]. Yet, the exact role of flavonoids in the pathology of the cardiovascular system remains unclear, which should encourage further research in this field [92].

## 5. Quercetin: Natural Source, Influence during Pregnancy, In Vitro and In Vivo Pharmacological Activities, Clinical Trials, Safety Profile

**Figure 1:**
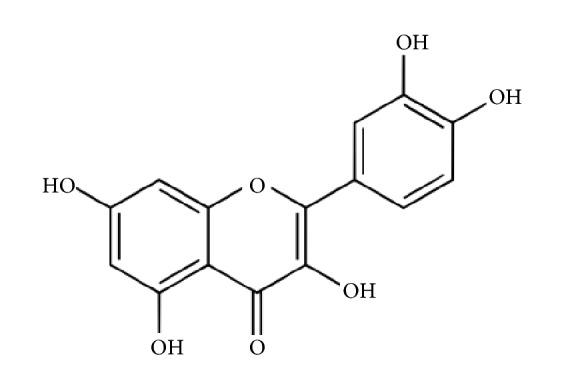
Chemical structure of quercetin.

Quercetin (3,3′,4′,5,7-pentahydroxyflavone) (Figure 1) is one of the most important representatives of flavonols, which is a subclass of flavonoids, and one of the most abundant flavonoids widely distributed in apples, berries, onions, tea, green vegetables, and herbs [93]. During the last years, several studies have reported beneficial effects of quercetin in cardiovascular diseases, such as hypertension, atherosclerosis, ischemia-reperfusion injury, or cardiotoxicity [78, 94, 95].

The mechanism of action of quercetin that is discussed below includes regulation of activity and structure of endothelial cells as well as anti-inflammatory and antioxidant properties, which are analyzed both in animal and human cell lines in models* in vivo* and in clinical trials. We believe that molecular and cellular mechanisms of action of quercetin may correspond with targets in PIH.

## 6. Quercetin Intake during Pregnancy

It is known that several flavonoids can cross the placenta and may be accumulated in fetal tissue [96, 97]. It was found that quercetin affects pregnancy in animal model [97–103]. According to Vanhees et al., when female mice were exposed to quercetin (302 mg/kg feed) from 3 days before conception until day 14.5 of gestation, no negative effects on placenta or fetal development were found [97]. An earlier study has shown that quercetin administrated to mice (333 mg/kg feed) did not have any teratogenic effects on the fetus [98]. Exposure to quercetin during pregnancy resulted in upregulation of genes involved in enzymatic antioxidant defense system of the liver of fetuses at day 14.5 of gestation [97]. Recently, Khaksary-Mahabady et al. [103] have observed a protective effect of quercetin (75 mg/kg) on histomorphometric changes of kidneys of fetuses in pregnant rats treated with all-transretinoic acid (a teratogenic metabolite). Interestingly, Toumi et al. [104] have shown that besides a protective effect with respect to hematological and behavioral anomalies in traumatized pregnant rats, quercetin (50 mg/kg per 6 days) may lastingly modulate behavior of their progeny. It was shown that quercetin administered prior to psychotraumatic stress alleviated signs of anxiety and hematological changes in pregnant rats without affecting the neonatal hematological status [104]. Moreover, it was demonstrated that a 6-week-quercetin treatment that started from day 3 postnatally improved significantly the memory ability of rats with hypoxic-ischemic brain damage [105]. A different study [99] has shown that quercetin reduced hydroxyurea-induced abnormal development of mouse embryos. Quercetin (66 mg/kg supplemented diet) significantly improved fetal skeletal maldevelopment that was provoked with high saturated fat diet perhaps in part due to antioxidant effects of quercetin in placenta. Similarly, Sistani Karampour et al. [101] have observed preventive effects of quercetin on theophylline-induced anomalies in embryos of pregnant female Wistar rats in groups where theophylline was used with quercetin (259 mg/kg, po and 100 mg/kg, ip, respectively) on the 9th and 10th day of gestation. Quercetin improved heart conditions, reduced malondialdehyde levels, and elevated glutathione peroxidase activity [101]. Cytoprotective effects of quercetin were also observed in rat fetal liver in a study where [102] concentrations of malondialdehyde were lower and catalase, superoxide dismutase, and glutathione peroxidase enzyme activities were higher in the group treated with ciprofloxacin (20 mg/kg) plus quercetin (20 mg/kg) for 21 days compared to the group after ciprofloxacin alone. Mahabady and Varzi [103] studied the prophylactic effect of quercetin (75 mg/kg) on teratogenic effects of caffeine (80 mg/kg), which was given intraperitoneally at 9th through 11th day of gestation of rats. It was observed that caffeine with quercetin significantly reduced incidence of cleft palate to 1.49% when compared to caffeine alone. The mean weight and length of animals' fetuses receiving quercetin were significantly increased when compared to other groups.

Zhao et al. [100] showed anti-abortive effect of quercetin in pregnant mice after injection of lipopolysaccharide (LPS). LPS induced embryo resorption in mice, while pretreatment with quercetin at a dose of 0.4 ml at days 4 to 7 decreased the abortion rate to 50.0% through modulation of maternal-fetal interface immunity balance, mainly by interleukin-10. In another study [106] it was observed that quercetin may inhibit proinflammatory cytokines production in pregnant mice, especially IL-6 and IL-8. Additionally, Braga et al. [107] investigated the effects of quercetin on the maternal reproductive capacity and fetal and placental development in diabetic rats. They showed that quercetin did not improve reproductive capacity, neither fetal nor placental development; however quercetin controlled glucose levels and promoted weight gain compared to untreated diabetic rats. Interestingly, it was observed in a different study [108] that quercetin at 10 mg/kg/day affects uterine morphology but not proliferation. Yet, at doses of 100 mg/kg/day quercetin induced significant stromal and glandular proliferation, which could predispose the uterus towards neoplastic development.

Yet another study showed that quercetin had protective effect on the injured endometrial cells model obtained from pregnant rats (5.5 days of gestation). The treatment with quercetin (50 *μ*mol/L for 24 h) increased expression of CYP1A1 and CYP2B1, as well as the contents of TNF-*α*, E_2_, and IL-6 in the* in vitro* model [109].

## 7. *In Vitro* Studies of Mechanism of Action

### 7.1. Animal Cells

**Figure 2:**
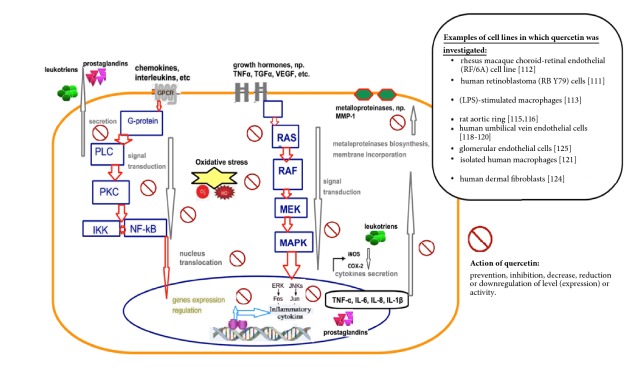
Examples of* in vitro* molecular action of quercetin (Q) in cited cellular experiments. Red arrows indicate the directions of interaction of intracellular proteins during signal transduction. Arrows marked in gray represent the direction of signaling in the cell or secretion beyond its expiration. The symbol of the swash in the wheel (red color) describes action of quercetin: prevention, inhibition, decrease, reduction, or downregulation of level (expression) or activity. Abbreviations: GPCR: G protein-coupled receptor; PLC: phospholipase C; PKC: protein kinase C; IKK: inhibitor of nuclear factor kappa-B kinase subunit beta; NF-*κ*B: nuclear factor-*κ*B; RAS, RAF, MEK, MAPK (p38 mitogen-activated protein kinase), ERK (extracellular signal-regulated kinase) pathway: Ras protein family members of small GTPases, involved in transmitting signals within cells (cellular signal transduction); Fos: protooncogenic transcription factor of the bZIP family, protooncogene; JUNKs (JNKs): c-Jun N-terminal kinases; Jun: one of the c-Jun N-terminal kinases; TNF-*α*: tumor necrosis factor *α*; IL-6, IL-8, IL-1*β*: inflammatory interleukins; COX-2: cyclooxygenase-2; iNOS: inducible NO synthase.

There are many studies that focus on anti-inflammatory activity of quercetin and its impact on growth factors in various* in vitro* models [109–112], which can be taken into account in therapy of pregnancy-induced hypertension (Figure 2). For example, Li et al. [112] showed that quercetin inhibited vascular endothelial growth factor (VEGF)-driven cell proliferation, migration, and tube formation of rhesus macaque choroid-retinal endothelial (RF/6A) cell line in a dose-dependent fashion: 22.7%, 31.5%, and 36.7% inhibition when treated with 10, 50, and 100 *μ*M of quercetin, respectively. Similarly, Song et al. [111] observed that quercetin inhibited the expression of VEGFR in a dose-dependent manner.

In a study on biomarkers of inflammatory process, quercetin was found to inhibit significantly the production of nitric oxide (NO), inducible NO synthase, interleukin-6, the nuclear translocation of nuclear factor-*κ*B (NF-*κ*B), extracellular signal-regulated kinase 1/2 (Erk1/2), and c-Jun N-terminal kinase (JNK) in LPS-stimulated macrophages [113]. Moreover, it inhibited MAPK/AP-1 and IKK/NF-*κ*B-induced inflammatory mediators [114]. Also, quercetin prevented angiotensin II-induced endothelial dysfunction [115] and inhibited protein kinase C activity and vascular superoxide production induced by endothelin-1 (in rat aortic ring) [116]. Moreover, Lee et al. [117] observed a protective effect of quercetin against arsenite-induced COX-2 expression (in rat liver epithelial cells).

### 7.2. Human Cells

Quercetin showed protective effects against inflammation in human umbilical vein endothelial cells via the downregulation of vascular cell adhesion molecule 1 (VCAM-1) [118]. Quercetin prevented lipid peroxidation induced by H_2_O_2_ and reduced the cytokine-induced cell-surface expression of VCAM-1 and E-selectin in cultured human endothelial cells [119, 120]. Treating isolated human macrophages with quercetin reduced inflammation by attenuating gene expression for tumor necrosis factor *α* (TNF-*α*) and interleukins (IL-6, IL-8, IL-1*β*) [121] and by inhibiting level of cyclooxygenase-2 (COX-2) and lipooxygenase (LOX) activities [122, 123]. Furthermore, the compound strongly inhibited induction of matrix metalloproteinase-1 (MMP-1, collagenase-1) and activation of extracellular signal-regulated protein kinase (ERK) and p38 mitogen-activated protein kinase (MAPK) (in human dermal fibroblasts) [124]. In glomerular endothelial cells, quercetin prevented asymmetric dimethylarginine (ADMA)-induced renal fibrosis by reducing TGF-*β* expression and ADMA-induced cell apoptosis mediated by the endoplasmic reticulum stress pathway [125].

## 8. *In Vivo* Studies

Several studies have stressed that quercetin showed free radical scavenger properties [126–128], which makes this flavonoid a well-established antioxidant that may be potentially used in prevention and treatment of cardiovascular disorders, even more as quercetin was reported to reduce oxidative damage to the brain, heart, and other tissues during ischemic reperfusion injury and exposure to compounds that induce oxidative stress [102, 129].

## 9. Antihypertensive Activity of Quercetin* In Vivo*

Quercetin has been shown to induce a progressive, dose-dependent, and sustained reduction in blood pressure when given chronically in several rat models of experimental hypertension, including spontaneously hypertensive rats (SHR), rats with hypertension induced by inhibition of NO synthase with N-nitro-L-arginine methyl ester (L-NAME), deoxycorticosterone acetate (DOCA)-salt hypertensive rats, rat model of renovascular hypertension with reduced blood flow to one kidney, and rat genetic model of metabolic syndrome [23–35, 130, 131]. Quercetin also prevented morphological and functional changes in the heart, vessels, and kidney [131]. The most important findings report that quercetin has fully reduced cytokine-stimulated expression of human C-reactive protein (CRP) and cardiovascular risk factors (murine serum amyloid A proteins, fibrinogen) in transgenic mice [98**]** and that it has led to decrease of inflammatory process in atopic dermatitis mouse model [114] and levels of TNF-*α*, IL-1*β*, IL-17, and MCP-1 in collagen-induced arthritis in various animal models [132]. Recent studies are summarized in Table 1.

## 10. Clinical Trials and Meta-Analysis

Several human clinical trials have been conducted so far to estimate the efficacy of quercetin intake in the reduction of risk for hypertension and in hypertension treatment [36–42]. Our literature review includes a recent meta-analysis of earlier clinical trials [43]. The Cochran collaboration did not perform comparative analysis of studies. The results are summarized in Table 2. A total of 7 clinical studies in which quercetin antihypertensive effect was estimated in adult patients (n = 169) were included in this review paper. Quercetin was used in a range of doses from 162 mg/day to 1095 mg/day from 7 days to 6 weeks. Five double-blind controlled crossover clinical trials concluded that supplementary intake of quercetin was able to lower blood pressure of hypertensive patients [37–40, 42]. In contrast, two clinical trials showed no effect of quercetin supplementation on blood pressure [36, 41].

Results of meta-analysis of clinical trials [43] have shown that the impact of quercetin on blood pressure was reported in a total of 7 trials (587 patients) with results revealing significant reductions in both systolic and diastolic blood pressure following quercetin supplementation at doses from 100 mg/day to 1000 mg/day from 4 to 10 weeks [93, 107, 133–137]. In summary, the randomized controlled trials show that a significant reduction in systolic and diastolic blood pressure may be expected with quercetin doses ≥500 mg/day and the effect is insignificant with doses <500 mg/day [43].

Proposed mechanism for such an effect of quercetin is still not completely understood but may include influence on the level of oxidative stress markers, endothelial function, vascular smooth muscle activity, modulation in cell signaling, and gene expression, which were all observed in some animal models [23, 25–28, 30, 31, 33, 34, 130]. Interestingly, in clinical trials, no reduction in stress markers was observed after quercetin administration, while* in vitro* quercetin showed such activity [36, 40, 42, 93]. Similarly, while quercetin has been shown to inhibit angiotensin-converting enzyme (ACE) function* in vitro* [138], clinical trials have observed no change in ACE activity [40]. Therefore, other targets and mechanisms of antihypertensive activity of quercetin should be considered.

Apart from chemical compounds obtained from natural sources, other active substances like acetylsalicylic acid (ASA) are candidates for consideration in the search for new pharmacological strategies for prevention and treatment of PIH. A systematic review and a meta-analysis showed that ASA treatment initiated early in pregnancy is an efficient method of reducing the incidence of PIH [139] and that low dose of ASA started in the first trimester in high-risk women may reduce the risk of PIH by up to 50% [140]. Actually, aspirin at dose of 75 mg is recommended by WHO [141] for the prevention of preeclampsia in women at high risk. However, randomized controlled clinical trials showed that aspirin initiated at >16 weeks of gestation was not associated with a risk reduction or a dose-response effect for severe PIH [142]. Moreover, a randomized placebo-controlled PREDO trial showed that aspirin at dose of 100 mg/day caused no statistically significant effect in preventing preeclampsia in high-risk women [143]. On the other hand, a yet different multicenter randomized controlled study reports that aspirin use was associated with greater rates of vaginal bleeding and that an appropriately powered randomized controlled trial is now required to address the efficacy and safety of universal low-dose aspirin in low-risk pregnancy [144]. Based on these studies, it can be concluded that the search for an active drug with a higher safety index continues.

## 11. Other Natural Compounds Tested

Apigenin has recently gained attention as an alternative flavonoid compound for prevention and treatment of PIH. Previously, Manolescu [145] has observed that apigenin exhibited an endothelial nitric oxide synthase (eNOS) stimulation effect and was able to inhibit HIF-1*α* expression as a factor causing placental hypoxia by blocking its interactions with HIF-1*α*-Hsp90 receptors. Recently, Hikmah et al. [146] have shown that an extract from* Apium graveolens* containing apigenin can prevent intrauterine growth restriction via suppression of antiangiogenic factors production in PIH animal model because it inhibited action of TNF*α* and HIF-1*α*.

Besides, it was shown that polyphenolic compounds exert anti-inflammatory properties and provide protection to the endothelial cells. For this reason, polyphenols may give some insight into their usefulness in PIH. A polyphenolic extract from* Theobroma cacao* has been reported to increase cells viability and reduce interleukine-6 and sVCAM-1 levels [147] as well as to increase nitric oxide and decrease endothelin-1 [148] in endothelial cells induced by plasma from PIH patients. Moreover, an extract of* Punica granatum* containing polyphenols (flavonoids and tannins) provided protection to the endothelial cells stimulated with severe PIH patients' plasma by suppressing production of proinflammatory cytokines (TNF-a and IL-6) and antiangiogenic factors (sFlt-1 and sEng) [149, 150]. Recently, a double-blind randomized and placebo-controlled clinical study showed that another polyphenolic compound, namely, epigallocatechin gallate (EGCG), increases the efficacy of nifedipine in patients with severe pregnancy-induced PIH [151]. Furthermore, an extract from* Camellia sinensis* (green tea) with polyphenols (catechins) showed anti-inflammatory properties during pregnancy of Wistar rats [152].

## 12. Potential Problems with the Use of Quercetin

Using quercetin as pharmacologic therapy in the form of dietary supplementation may encounter few problems. Firstly, bioavailability of quercetin is generally poor and it is affected by numerous factors [78, 153, 154]. Moreover, bioavailability of quercetin is very variable. Review of Harwood et al. [155] showed that in humans total plasma quercetin levels (i.e., quercetin, quercetin glycosides, glucuronides, and sulfates) between 29 and 248 ng/ml were attained following ingestion of a single meal consisting of quercetin-rich foods (50 mg quercetin). Another study showed that healthy subjects provided with 100 mg of radiolabeled quercetin absorbed up to 53% of the total dose [156]. Quercetin dietary supplement formulations contain aglycone whereas food items contain mainly quercetin glycosides [157]. It is well known that after oral ingestion quercetin glycosides are physiologically hydrolyzed by glucosidases in the intestine and next quercetin aglycones passively diffuse through the intestinal epithelial barrier. However, quercetin glycosides may also be absorbed directly via the intestinal sodium-dependent glucose transporter-1 (SLGT1) [157, 158]. This type of absorption provides greater intestinal uptake of quercetin glucoside [154, 159]. Both animal and human studies have shown that oral administration of quercetin yields as much as 60% of absorption [155]. It needs to be highlighted that the optimum intake of flavonoids from supplements has not been defined yet because a number of exogenous and endogenous factors modulating their bioavailability affect their vascular function [78]. Moreover, according to Terao [78] the low bioavailability of quercetin supplement may be partly attributed to its poor dispersion capacity in the digestive tract.

Presently, chemical modification of quercetin has led to new semisynthetic derivatives with improved bioavailability and solubility. These new compounds are still being investigated [160–162].

Another problem is that in the* in vitro* models it is the metabolites of quercetin that should be studied, not the parent compound. In human intestinal compartment quercetin is converted by phase II enzymes during the absorption process and, thus, it is the conjugated metabolites that are present in circulation [158]. Therefore, researchers should be focused on glucuronide/sulfate conjugates of quercetin when estimating their vascular effects using* in vitro* and* in vivo* models.

Extrapolating results from* in vitro* to* in vivo* is yet another problem as the heterogeneity of human organism cannot be simply replicated* in vitro*. Moreover, according to Avila-Galves et al., the use of a single cell line culture should be avoided [163]. The use of multiple cell lines with different features or different cell types in cocultures is postulated instead. Yet, such the procedure is far from easy. For this reason many studies have several limitations.

It was shown that quercetin is a multidrug resistance (MDR) modulator and thus it is a potential chemosensitizer as it can inhibit P-gp activity or it can decrease its expression and can interact with the ATP-binding site or the substrate-binding site [164]. Consequently, on the basis of several studies, it should be realized that quercetin may interact with medicines such as anticancer drugs [165].

## 13. *In Vitro* and* In Vivo* Toxicity and Drug Interaction

According to International Agency for Research on Cancer of WHO [166] quercetin is not classifiable as to its carcinogenicity to humans (Group 3). Review of animal studies has shown that quercetin may increase drug bioavailability of many drugs including irinotecan, etoposide, tamoxifen, paclitaxel, doxorubicin (anticancer drugs), digoxin (drug used in heart failure), verapamil and diltiazem (calcium channel blockers used in the treatment of hypertension, angina pectoris, and some types of heart arrhythmia), valsartan (antihypertensive drug), ranolazine (drug to treat angina pectoris), and paracetamol [167].

## 14. Safety Data from Clinical Trials

Among the numerous published human intervention studies, adverse effects following supplemental quercetin intake have been rarely reported and any such effects were mild in nature [167]. So far no adverse events were reported in clinical trials where participants used quercetin at doses of 500 mg per day for 4–8 weeks [137, 168], 730 mg for 4 weeks [93], or 1000 mg for 5 days, for at least 2 weeks or for 12 weeks [169]. Moreover, any adverse effects were associated with a single oral dose beyond 4 g [170]. After intake of 1000 mg per day for 1 month by patients with chronic pelvic pain syndrome, one patient developed headaches [171]. According to the US Food and Drug Administration quercetin has a generally recognized safe status (GRAS) [172]. Considering the seasonal supply of food extracts of flavonoids, the Recommended Dietary Allowance of total flavonoids might be between 250 and 400 mg/day [173, 174]. However, in dietary supplements, recommended daily doses of quercetin aglycone usually reach up to 1000 mg (most commonly 500 mg) [167].

Quercetin in humans caused a reduced bioavailability of midazolam (sedative) and talinolol (antihypertensive drug), and an increased bioavailability of cyclosporine, pravastatin (cholesterol-lowering drug), and fexofenadine (antihistamine drug) [167]. Furthermore, it is considered that patients with a kidney dysfunction may be a potential risk group for a long-term quercetin supplementation at high doses (potential nephrotoxic effects of quercetin) [167].

## 15. Conclusion

In summary, review of results of pharmacological studies shows that quercetin possesses antihypertensive activity* via* various mechanisms of action. While most studies have been carried out in in vitro and animal models, the antihypertensive mechanisms of quercetin in clinical studies remain somewhat elusive. Moreover, the influence of pure quercetin per se on pregnancy-induced hypertension has not been investigated. Nevertheless, various studies have shown that quercetin has a beneficial effect on vascular endothelial function and exhibits antioxidative and anti-inflammatory activity on cellular and tissue level. On the other hand, quercetin can affect the development of embryo, fetus, and placenta during pregnancy in animal model. Cytoprotective properties with regard to the fetus were observed in numerous studies. It was shown that quercetin did not have teratogenic and abortive effect on the fetus and it is generally recognized as safe.

All in all, these results allow formulating a proposition that quercetin be a new potential plant-origin drug in the treatment of PIH. Yet, for such potential to be realized, multilevel and complex pharmacological studies are needed. Future investigation should be focused on key factors among other inflammatory mediators and growth factors such as vascular endothelial growth factor, placental growth factor, and basic fibroblast growth factor, all involved in pathogenesis of pregnancy-induced hypertension.

## Figures and Tables

**Table 1:** Recent experimental pharmacological *in vivo* studies on quercetin.

**Flavonoid **	**Animal model**	**Dose**	**Main results**	**Ref. **
quercetin	Sprague-Dawley rats; N^*ω*^-nitro-L-arginine methyl ester (L-NAME) treatment to increase in blood pressure (BP)	4 g quercetin/kg diet for four days	(i) increase NOS activity (after quercetin in the absence of L-NAME), (ii) reduction in heart glutathione oxidation ratio (after quercetin with L-NAME), (iii) decrease in systolic BP (after quercetin with L-NAME)	[23]

quercetin	two-kidney one-clip (2K1C) hypertensive Wistar rats	10 mg kg^−1^ b.w. per day Intragastrically for three weeks	(i) reduction in systolic BP, (ii) reduction the hypertrophic remodeling in hypertension by decrease: (a) the number of vascular smooth muscle cells (b) excessive production of superoxide anion in hypertensive aorta (c) MMP (matrix metalloproteinase) activity and MMP-2 expression	[24]

quercetin	Wistar rats; cobalt chloride-induced hypertension;	50 mg kg^−1^; cocktail of 50 mg kg^−1^ quercetin + 100 mg kg^−1^ vit. C b.w. per day intragastrically for 14 days	In vivo: quercetin and/or vitamin C significantly decreased in the systolic, diastolic and mean arterial blood pressure (MAP) of rats from hypertensive to normotensive values Ex vivo: quercetin and/or vitamin C significantly reduced the level of oxidative stress markers (hydrogen peroxide, malondialdehyde), increased in the activities of antioxidant defence system components (glutathione peroxidase, reduced glutathione, protein thiol, and non-protein thiol)	[25]

quercetin	Sprague-Dawley rats; pulmonary arterial hypertension;	100 mg kg^−1^ b.w. per day intragastrically for 14 days	In vivo: Quercetin inhibited proliferation and increased the apoptosis of pulmonary artery smooth muscle cells In vitro: Quercetin increased cyclin D1 protein levels, decreased the protein expression of cyclin B1 and Cdc2, altered the Bax/Bcl-2 ratio, reduced MMP2, MMP9, CXCR4, integrin *β*1, integrin *α*5 expression	[26]

quercetin	Wistar rats; pulmonary arterial hypertension	10 mg kg^−1^ b.w. per day, per os for 10 days	In vivo: quercetin significantly restored the decrease in Kv currents, the upregulation of 5-HT2A receptors and reduced the Akt and S6 phosphorylation. In vitro: quercetin induced pulmonary artery vasodilator effects, inhibited pulmonary artery smooth muscle cell proliferation and induced apoptosis	[27]

quercetin	spontaneously hypertensive (SHR) rats	10 mg kg^−1^ b.w. per day, per os or i.p. injection for 5 weeks	Reduction in (i) systolic BP, heart rate (ii) NADPH oxidase activity (p.o.), without effect after i.p. administration (iii) protein expression of the NADPH subunits p47, NOX1, NOX4	[28]

quercetin	Sprague-Dawley rats; monocrotaline induced pulmonary arterial hypertension	100 mg kg^−1^ b.w. per day, per os for 21 days	Reduction in (i) mean pulmonary artery pressure, (ii) monocrotaline induced increase in wall thickness and wall area, (iii) monocrotaline induced increase in expression of the pulmonary artery tissues proliferating cell nuclear antigen (PCNA)	[29]

quercetin	spontaneously hypertensive (SHR) rats;	2, 10 or 25 mg kg^−1^ b.w. per day, per os for 7 days	(i) decrease the mean arterial pressure (10 or 25 mg kg^−1^), (ii) increase the sensitivity of parasympathetic component of the baroreflex, (iii) decrease the serum oxidative stress	[30]

quercetin	SHR rats;	10 mg kg^−1^ per day intragastrically for 4 weeks	In vivo: (i) progressive reduction in systolic BP (ii) reduction (−14%) in mean arterial BP and heart rate, (iii) reduction MDA levels in plasma Ex vivo: (i) increase in the endothelium dependent relaxation induced by acetylcholine (aorta)	[31]

quercetin	SHR rats;	1.5 g quercetin/kg^−1^ diet for 5 or 11 weeks	(i) the lack of efficacy of quercetin in parameters of hypertension, vascular dysfunction, cardiac hypertrophy	[32]

quercetin	SHR rats;	10 mg kg^−1^ per day intragastrically for 5 weeks	In vivo: (i) reduction in systolic (by18%), diastolic (by 23%) and mean (by 21%) arterial BP; (ii) reduction of heart rate (by 12%); (iii) reduction in parameters: the heart weight index (HW/BW), the left ventricular weight index (LVW/BW) and the kidney weight index (KW/BW); (iv) tendency to increase the lumen diameter (LD) and to reduce the media thickness (MT) Ex vivo: (i) increasing the vasodilation induced by acetylcholine (aorta);	[33]

dihydroquercetin	SHR rats	20 mg kg^−1^ per day intragastrically for 6 weeks	Decrease of (i) blood pressure, BP (by 11%), (ii) left ventricular mass index, LVMI (by 2%), (iii) blood viscosity at shear rate (by 7-10%)	[26]

dihydroquercetin	SHR rats	50 mg kg^−1^ per day intragastrically for 6 weeks	Increase in: (i) the mean capillary diameter (by 11%), (ii) capillary network density (by 23%), (iii) share of capillaries passable for erythrocytes (by 42%); Improvement of microcirculation in the cerebral cortex	[34]

dihydroquercetin	SHR rats; normotensive WKY rats	100 and 300 *μ*g kg^−1^ per day intragastrically for 2 weeks	(i) no effect on BP or ACE activity in SHR rats (two doses); (ii) no effect on BP in WKY rats (max. dose) (iii) reduction of ACE activity in WKY rats (max. dose)	[35]

**Table 2:** Recent randomized controlled trials of quercetin-based formulations and hypertension.

**Flavonoid/preparation**	**Volunteers' data **	**Trial data **	**Main results **	**Ref. **
Quercetin in extract (capsules contained onion skin extract powder (132 mg) with 54 mg quercetin per capsule)	n = 22, male (11), female (11), overweight-to-obese hypertensive patients	randomized, double-blind, controlled, crossover meal study; dosage: long-term supplementation (162 mg/day quercetin) with 6-week treatment periods	(i) no effect of treatment on blood pressure (BP), heart rate, or any biomarker of endothelial function (ii) lack of effect on antioxidant activity and oxidative stress,	[36]

Provex CV (combination of extracts from grape seed and skin (330 mg), green tea (100 mg), resveratrol (60 mg) and a blend of quercetin, ginkgo biloba and bilberry (60 mg))	n = 20, age: 18 - 65 years, male, female, patient with stage 1 hypertension (metabolic syndrome)	double-blinded, placebo-controlled, randomized, crossover trial; dosage: supplementation with 28-day treatment periods (330 mg Provex CV/day) ClinicalTrials.gov Identifier: NCT01106170	(i) reduction in diastolic pressure (ii) unchanged systolic pressure (iii) trend to reduction in arterial pressure (iv) potentiation eNOS activation and nitric oxide production	[37]

Pure quercetin-3-glucoside	n = 37, age: 40 - 80 years male, female, healthy (pre)hypertensive participants	double-blind, randomized, placebo-controlled crossover trial; dosage: 160 mg of quercetin-3-glucoside in capsule daily for a period of 4 weeks ClinicalTrials.gov Identifier: NCT01691404	(i) decrease of plasma sE-selectin,	[38]
			(ii) no effect of treatment on other biomarkers of endothelial dysfunction (sICAM-1, sVCAM-1, and MCP-1), (iii) decrease IL-1b level, (iv) no effect of treatment on other biomarkers of inflammation (CRP, serum amyloid A (SAA), TNF-a, IL-6, IL-8, and sICAM-1), (v) decrease the score for inflammation, (vi) no effect on flow-mediated dilation, insulin resistance, or other CVD risk factors	[39]

quercetin from onion skin extract	n=70 patients with pre-hypertension and stage I hypertension, men, women (25–65 years)	double-blinded, placebo-controlled, randomized cross-over trial dosage: 162 mg/d quercetin per 6 weeks 3x54 mg quercetin per one gelatin capsules	(i) decrease day-time and night-time systolic BP (ii) no changes in vasoactive biomarkers: endothelin-1, soluble endothelial-derived adhesion molecules, asymmetric dimethylarginine, angiotensin-converting enzyme activity, endothelial function, parameters of oxidation, inflammation, lipid and glucose metabolism	[40]

quercetin	n=15 men (9), women (6) (26 ± 5 years)	double-blind, placebo-controlled, randomized crossover study dosage: 200 and 400 mg of quercetin per 3 weeks	(i) 400 mg increased the plasma levels of glutathione (ii) no changes in systolic and diastolic blood pressure after 2 and 5h post ingestion of 200 and 400 mg quercetin	[41]

quercetin	n = 12 of stage 1 hypertensive men (41 ± 12 years) n = 5 of normotensive men (24 ± 3 years)	double-blind, placebo-controlled, crossover trial dosage: 1095 mg of quercetin in tablet per 7 days	hypertensive men: (i) decrease in systolic, diastolic, and mean BP at 10 hours post quercetin ingestion (ii) no effect on plasma ACE activity, NO metabolites, ET-1, and the ratio of ET-1:NO metabolites, relative flow-mediated dilation (FMD) normotensive men: (i) no effect of supplementation on BP, heart rate, ACE activity	[42]

quercetin	n = 587 participants (299 in quercetin group; 288 in control group)	7 placebo-controlled randomized controlled trials (between 1998 and 2014). Ranges of doses from 100 to 1000 mg/ day of quercetin per 4 and 10 weeks	conclusion of meta-analysis: (i) statistically significant effect of quercetin supplementation in the reduction of blood pressure (systolic and diastolic BP), possibly limited to, or greater with dosages of >500 mg/day, (ii) oral quercetin administration was safe and well tolerated,	[43]
